# A novel characterization of posterior keratoconus using anterior segment optical coherence tomography in an infant: a case report

**DOI:** 10.1186/s12886-015-0139-3

**Published:** 2015-11-04

**Authors:** Nisha Garg, Ta C. Chang, Bibiana Jin Reiser, Kara M. Cavuoto

**Affiliations:** Department of Ophthalmology, Bascom Palmer Eye Institute, University of Miami Miller School of Medicine, 900 NW 17th St, Miami, FL 33136 USA; Children’s Hospital Los Angeles, 4650 Sunset Blvd., Los Angeles, CA 90027 USA; University of Southern California Eye Institute, 1450 San Pablo Street, Los Angeles, CA 90033-4682 USA

**Keywords:** Posterior keratoconus, Corneal opacity, Infant, Anterior segment optical coherence tomography

## Abstract

**Background:**

Posterior keratoconus is a rare cause of a corneal opacity in an infant. It is characterized by thinning of the posterior cornea without ectasia of the anterior cornea. Imaging of this condition with anterior segment optical coherence tomography (AS-OCT) has not been reported in the literature.

**Case presentation:**

A six week old African-American male presented with a congenital corneal opacity of the right eye. He underwent an examination under anesthesia in which photography and AS-OCT were performed. AS-OCT confirmed the diagnosis of posterior keratoconus. The patient subsequently underwent an optical iridectomy for visual development.

**Conclusion:**

AS-OCT is a useful tool in cases when a child presents with a corneal opacity of unknown or unclear etiology. In our patient, AS-OCT showed the classic description of central corneal thinning seen in this condition. Additionally, it revealed an associated detached Descemet membrane, a feature which has not been previously described in posterior keratoconus.

## Background

Posterior keratoconus is a rare, non-progressive corneal condition first described by T. Harrison Butler in 1930 as a “small basin-like depression” in the posterior surface of the cornea [1]. Also known as keratoconus posticus, it is characterized by thinning of the posterior cornea without ectasia of the anterior cornea. It presents as a corneal opacity and is generally considered a developmental abnormality, however it can also be acquired after ocular trauma [2]. This anomaly can be divided into two groups: generalized posterior keratoconus, in which there is uniform corneal steepening, and circumscribed posterior keratoconus, in which there is a localized posterior corneal indentation.

The histopathology of the abnormal cornea includes disorganization of the basal epithelium and basement membrane, fibrous replacement of Bowman layer, thinned stroma with scarring and irregular arrangements of central collagen lamellae and variable structural changes in Descemet membrane [3, 4]. The latter includes thinning with small breaks, a multilaminar configuration, abnormal anterior banding and localized posterior excrescences [3, 4]. Topographic analysis of the cornea has further shown that there are in fact anterior surface changes in posterior keratoconus, including central steepening in the area of the posterior corneal depression with gradual paracentral flattening [5]. Ultrasound biomicroscopy has been used to show the local anterior bulging of the posterior corneal surface, thinning of the stroma and enhanced stromal reflectivity corresponding to the stromal opacity seen in circumscribed posterior keratoconus [6].

This is the first characterization of posterior keratoconus using anterior segment optical coherence tomography (AS-OCT). The diagnosis of posterior keratoconus is typically made by visualizing the increased posterior corneal curvature during slit lamp examination, a difficult task in a very young child. However making the diagnosis is exceptionally important as any central corneal opacity portends a poor visual outcome, especially if it occurs during the critical period and/or is unilateral. New tools like AS-OCT have proven useful in distinguishing this diagnosis from other types of severe anterior segment dysgenesis, such as Peter anomaly. This study also revealed a detached Descemet membrane, which to date has never been reported in this condition. We speculate that this may be due to a decrease in the hydrostatic pressure in the area of the depression or an abnormality in the attachment between the neural crest-derived primordial epithelium and the surface ectoderm.

## Case presentation

**Fig. 1:**
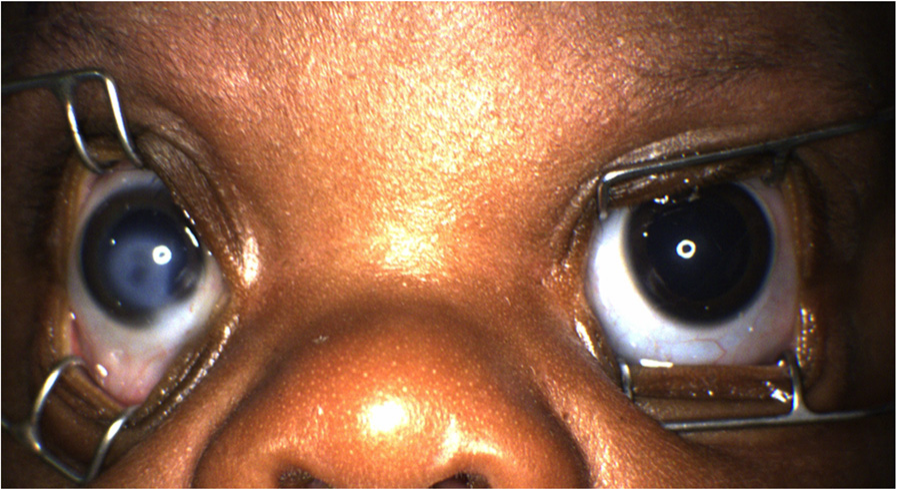
Photograph from examination under anesthesia demonstrating a corneal opacity of the right eye and a clear cornea of the left eye

A 6-week old African-American male presented for an ophthalmologic evaluation for a right corneal opacity noted at birth by a neonatologist (Fig. 1). He was born at 38 weeks gestation by spontaneous, unassisted vaginal delivery, and at the time of delivery the mother had a yeast infection and chlamydia, which were being treated. Family history was significant for adult-onset glaucoma in the maternal grandmother. The history of maternal infection and family history were felt to be non-contributory toward the condition. Referral to the ophthalmology clinic was delayed due to social issues. On examination, the patient blinked in response to light in the left eye but had questionable light perception in the right eye. Portable slit lamp examination of the right eye showed central corneal thinning with an opacity over the pupil. The anterior chamber and lens were poorly visualized due to the opacity. The fundus examination was limited, however there was a minimally intact red reflex after dilation. Examination of the left eye was normal.

**Fig. 2:**
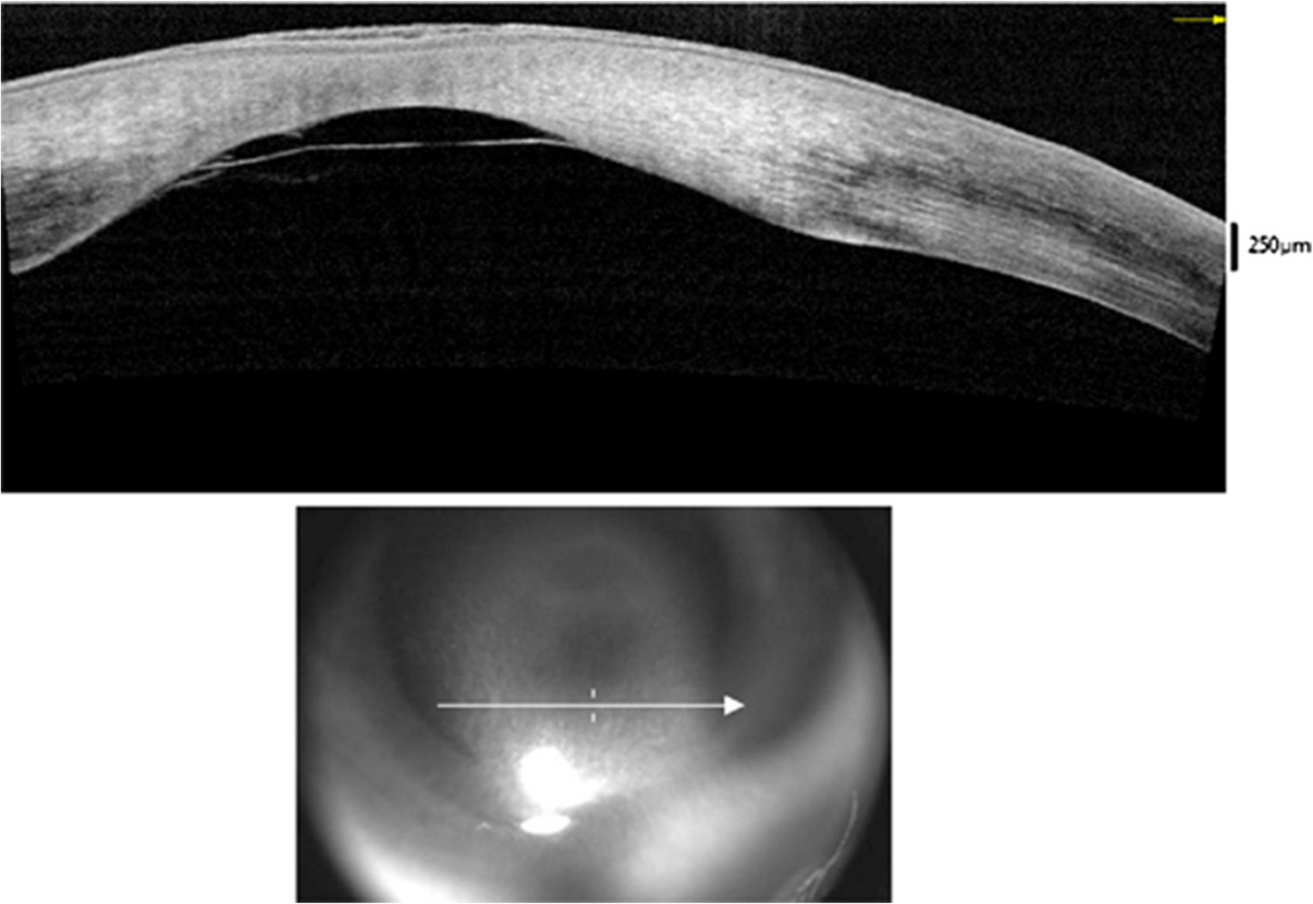
Anterior segment optical coherence tomography (AS-OCT) of the right eye showing central corneal thinning with a central Descemet detachment and an absence of corneal-lenticular adhesion

Examination under anesthesia was conducted at seven weeks of age. Intraocular pressures obtained by Tono-pen (Tono-Pen XT applanation tonometer, Reichert Inc., Buffalo, NY, USA) were 11 mm Hg in the right eye and 10 mm Hg in the left eye. Anterior segment examination of the right eye showed circumferential posterior embryotoxon and a dense central leukoma. The iris appeared to be slightly atrophic around the pupillary border, and the lens was mostly clear except for cortical vacuoles in the periphery. A slit-lamp examination of the right cornea showed posterior central thinning. Pachymetry of the right eye was obtained superiorly (693 microns), temporally (696 microns), and paracentrally in the superior portion of the leukoma (1073 microns). Attempts to obtain pachymetry over the corneal thinning were not successful due to poor applanation. Pachymetry of the left eye was 656 microns centrally. Examination of the left eye was unremarkable except for posterior embryotoxon seen in the infratemporal quadrant and two clusters of peripheral cortical vacuoles in the lens. Echography showed normal posterior poles bilaterally. Tests for herpes simplex virus and chlamydia were negative per the external records from the obstetrician. AS-OCT of the right eye revealed central corneal thinning with a central Descemet’s detachment and no corneo-lenticular adhesion (Fig. 2). The diagnosis of posterior keratoconus was made and the patient underwent optical iridectomy of the right eye. After optical iridectomy, both anatomic and visual development success were obtained as a red reflex was established and the patient developed fixation behavior.

## Conclusions

Posterior keratoconus is a rare corneal condition characterized by thinning of the posterior cornea without ectasia of the anterior cornea. It presents as a corneal opacity and is generally considered a developmental abnormality. It is typically non-progressive, however the opacity can have a significant impact on visual development. Our case demonstrates the utility of AS-OCT in differentiating the causes of a congenital corneal opacities and confirming the diagnosis of a rare disease entity such as posterior keratoconus. Given the pattern of centrally thickened region without a gradual thinning towards the center of the cornea, a non-keratoconic ectasia would also be in the differential diagnosis. Additionally, AS-OCT revealed an associated detached Descemet membrane, a feature which has not been previously described in this condition.

## Consent

Written informed consent to obtain and publish images was obtained from the patient’s legal guardian at the time of the examination under anesthesia. A copy of the consent is available for review by the Editor of this journal.

## Abbreviations

AS-OCT: Anterior segment optical coherence tomography
mm Hg: Millimeters of mercury
